# Consensus Report of Group 3 of the 1st Global Consensus for Clinical Guidelines for the Rehabilitation of the Edentulous Maxilla: Advanced Diagnostic Imaging, Augmentation Techniques, and Management of Complications

**DOI:** 10.1111/clr.70079

**Published:** 2026-02-24

**Authors:** Joseph Fiorellini, Guo‐Hao Lin, Isabella Rocchietta, Sean Mojaver, Tara Aghaloo, Kang‐Min Ahn, Bilal Al‐Nawas, Maurício Araújo, Jamil Awad Shibli, James Chow, Marcelo Faveri, Tobias Fretwurst, Markus Hürzeler, Darnell Kaigler, Fouad Khoury, Sungtae Kim, Marcel F. Kunrath, Hong‐Chang Lai, Michel R. Messora, Robert Sader, Muhammad H. A. Saleh, Junyu Shi, Simon Storgård Jensen, Tiziano Testori, István Urban, Yiqun Wu, Alvin Zad, Hom‐Lay Wang, Christer Dahlin

**Affiliations:** ^1^ Department of Periodontics University of Pennsylvania School of Dental Medicine Philadelphia Pennsylvania USA; ^2^ Division of Periodontology, San Francisco (UCSF) School of Dentistry University of California San Francisco California USA; ^3^ Department of Periodontics & Oral Medicine University of Michigan School of Dentistry Ann Arbor Michigan USA; ^4^ Department of Periodontology UCL Eastman Dental Institute London UK; ^5^ Department of Biomaterials University of Gothenburg, Sahlgrenska Academy Gothenburg Sweden; ^6^ Section of Oral & Maxillofacial Surgery, Los Angeles (UCLA) School of Dentistry University of California Los Angeles California USA; ^7^ Department of Oral & Maxillofacial Surgery, Asan Medical Center University of Ulsan College of Medicine Seoul South Korea; ^8^ Department of Oral, Maxillofacial & Plastic Surgery University Medical Center of Johannes Gutenberg University Mainz Mainz Germany; ^9^ Periodontology State University of Maringá (Universidade Estadual de Maringá – UEM) Paraná Brazil; ^10^ Department of Periodontology & Oral Implantology University of Guarulhos (UNG) Guarulhos Brazil; ^11^ Faculty of Dentistry University of Hong Kong Hong Kong SAR China; ^12^ Shanghai Jiao Tong University Shanghai China; ^13^ Department of Oral & Craniomaxillofacial Surgery/Center for Dental Medicine University Medical Center Freiburg Freiburg Germany; ^14^ Department of Oral & Maxillofacial Surgery Charité – Universitätsmedizin Berlin Berlin Germany; ^15^ Albert‐Ludwigs‐Universität Freiburg (University of Freiburg) Germany; ^16^ Department of Oral & Maxillofacial Surgery University of Münster Münster Germany; ^17^ Department of Periodontology Seoul National University School of Dentistry Seoul South Korea; ^18^ School of Health & Life Sciences Department of Dentistry Pontifical Catholic University of Rio Grande Do Sul (PUCRS) Porto Alegre Brazil; ^19^ Department of Implant Dentistry Shanghai Ninth People's Hospital, Shanghai Jiao Tong University School of Medicine Shanghai China; ^20^ Department of Oral & Maxillofacial Surgery and Periodontology, Ribeirão Preto School of Dentistry University of São Paulo (FORP/USP) Brazil; ^21^ Department of Oral, Craniomaxillofacial & Facial Plastic Surgery/Dental Institute Carolinum Goethe University Frankfurt, University Hospital Frankfurt Germany; ^22^ Department of Odontology & Rigshospitalet, Department of Oral & Maxillofacial Surgery University of Copenhagen Copenhagen Denmark; ^23^ IRCCS Galeazzi–Sant'ambrogio Hospital & Department of Biomedical, Surgical and Dental Sciences University of Milan Milan Italy; ^24^ Faculty of Dentistry University of Szeged Szeged Hungary; ^25^ Department of Second Dental Center Shanghai Ninth People's Hospital, Shanghai Jiao Tong University School of Medicine Shanghai China

**Keywords:** dental implants, edentulous maxilla, implant overdentures, implant‐supported fixed prostheses dentures, sinus floor augmentation

## Abstract

**Objectives:**

The 1st Global Consensus for Clinical Guidelines (GCCG) in Implant Dentistry introduced an evidence‐based, patient‐centered framework for rehabilitating the edentulous maxilla. Working Group 3 aimed to develop clinical recommendations on advanced diagnostic imaging, soft and hard tissue augmentation, and management of complications.

**Materials and Methods:**

Recommendations were developed following the S2k‐level guideline framework of the AWMF and a structured nominal group technique. The evidence base included two systematic reviews on clinician‐reported outcomes (ClinROs) and patient‐reported outcomes (PROs), supplemented by single‐round international surveys of expert clinicians, patients, and cross‐disciplinary experts. Survey content addressed diagnostics, treatment planning, clinical procedures, and maintenance care. Draft recommendations were discussed at the in‐person consensus meeting in Boston (June 16–18, 2025) and finalized through anonymous plenary voting. Consensus thresholds were predefined as ≥ 75% but ≤ 95% agreement for consensus and > 95% agreement for strong consensus.

**Results:**

Working Group 3 formulated four clinical recommendations spanning advanced diagnostic imaging, augmentation techniques, and complication management. All four recommendations reached consensus. Voting participation per recommendation ranged from 68 to 88 (mean 79) out of a possible 105 participants.

**Conclusions:**

The Working Group 3 consensus statements offer practical guidance on surgical procedures and complication management in the rehabilitation of the edentulous maxilla. These recommendations are grounded in current evidence, interdisciplinary clinical practice, and patient perspectives. Furthermore, important evidence gaps, particularly in standardized patient‐reported outcome measures (PROMs), long‐term outcomes, and maintenance protocols, highlight key priorities for future clinical research.

## 1 Introduction

The 1st Global Consensus for Clinical Guidelines (GCCG) in Implant Dentistry introduced an innovative, evidence‐based framework for consensus‐building in the field. Centered on the rehabilitation of the edentulous maxilla, the initiative aimed to develop practical, patient‐centered clinical recommendations. The Working Group 3 examined key aspects of diagnostic imaging and digital planning, hard and soft tissue augmentation techniques, maintenance protocols, and the management of complications associated with implant‐based rehabilitation. These domains address challenges encountered during the rehabilitation of the severely resorbed maxilla, such as the need for bone augmentation, soft tissue grafting, maintenance care, and management of complications.

In preparation for the consensus conference, two systematic reviews (Sabri et al. 2026; Saleh et al. 2026) were conducted, and single‐round international surveys were utilized to gather evidence on clinician‐reported outcomes (ClinROs) and patient‐reported outcomes (PROs), as well as their outcome measures (CROMs and PROMs), relevant to the interventions (Brunello, Lin, et al. 2026; Lin, Brunello, et al. 2026). The consensus meeting (June 16–18, 2025 in Boston, Massachusetts, USA) followed a structured Nominal Group Technique. Each recommendation was drafted on the basis of systematic review findings and patient and cross‐disciplinary expert survey data (Lin, Brunello, et al. 2026; Sabri et al. 2026; Saleh et al. 2026), and then refined through individual review, prioritization voting, and iterative small‐group discussion. Consensus thresholds were defined as follows: strong consensus (> 95% agreement), consensus (≥ 75% but ≤ 95% agreement), and no consensus (< 75% agreement) among the voting participants. All recommendations were subsequently confirmed during the plenary session through formal voting.

What follows is the Working Group 3 consensus report, outlining the agreed‐upon guidelines for diagnostic tools, treatment planning and procedures, maintenance care, and the management of complications in the rehabilitation of the edentulous maxilla. Each section integrates the best available evidence (from systematic reviews and key literature), patient and cross‐disciplinary expert survey results, recommended outcome measures, and commentary on alignment or divergence from existing literature (Brunello, Lin, et al. 2026; Brunello, Strauss, et al. 2026; Lin, Brunello, et al. 2026; Sabri et al. 2026; Saleh et al. 2026; Strauss et al. 2026).

The Working Group 3 examined several critical domains in the clinical workflow for edentulous maxilla rehabilitation, focusing on interventions that can improve implant therapy outcomes in advanced cases. These domains included:

- **Diagnostics and Treatment Planning:** The routine use of volumetric imaging (cone‐beam computed tomography [CBCT] or computed tomography [CT] scans) for preoperative assessment and digital planning of implant placement in the edentulous maxilla. This also encompasses virtual treatment planning tools to optimize implant positioning with the prosthetic end result in mind.
- **Treatment Procedures—Hard Tissue Augmentation:** Techniques for augmenting deficient maxillary bone, such as guided bone regeneration (GBR) with membranes or meshes and sinus floor elevation (sinus lift). Special emphasis was placed on approaches to enhance graft stability, such as fixation of barrier membranesand ridge augmentation.
- **Treatment Procedures—Soft Tissue Augmentation:** Approaches to increase the width of keratinized mucosa around implants (e.g., free gingival grafts or connective tissue grafts), performed either at implant placement or at second‐stage surgery, to improve peri‐implant soft tissue health, facilitate oral hygiene, and enhance patient comfort.
- **Maintenance Protocols:** Long‐term supportive care strategies after full‐arch rehabilitation, including schedules for professional follow‐up and cleaning, patient education in oral hygiene, and monitoring protocols (e.g., periodic probing and radiographs) to prevent or detect peri‐implant complications at an early stage.
- **Management of Complications:** Intraoperative and perioperative complication management was addressed, with particular emphasis on the handling of common surgical challenges such as Schneiderian membrane perforation during sinus augmentation. Guidelines were developed to ensure predictable management of these complications, supporting successful outcomes even in the face of unexpected events.

Each of these domains was addressed by systematic reviews (Sabri et al. 2026; Saleh et al. 2026) and expert opinion, recognizing that socioeconomic and logistical factors (e.g., availability of specialist surgical expertise, treatment setting, and material resources) may influence the selection and implementation of interventions.

## 2 Preamble

This consensus applies to patients with a fully edentulous maxilla—or those for whom extraction of all remaining maxillary teeth is indicated—who are candidates for implant‐supported prosthetic rehabilitation. As in all clinical decision‐making, preservation of natural dentition remains the primary objective; edentulation of the maxilla is a significant intervention and falls outside the scope of this report. Every effort should be made to maintain teeth when clinically feasible, given the biological and functional advantages of natural dentition. Full‐arch implant rehabilitation should be considered only when tooth preservation is no longer viable, and even then, treatment must follow a comprehensive evaluation of patient‐specific factors such as patients' ability to perform adequate personal oral hygiene.

The prototypical patient addressed by the Working Group 3 is an adult with a completely edentulous maxilla (or one anticipated to become edentulous following planned extractions) who seeks, or would benefit from, implant‐supported rehabilitation. These patients present with varying degrees of ridge resorption (Saleh et al. 2026), ranging from minimal residual bone volume—requiring advanced surgical strategies such as those outlined by Working Group 2 (e.g., zygomatic or short implants) (Strauss et al. 2026)—to sufficient bone that allows for the placement of standard‐length implants. Patients in the latter category may also benefit from adjunctive procedures such as soft tissue augmentation (Covani et al. 2013; Lin et al. 2013) to optimize peri‐implant tissue health (Table 1).

**TABLE 1:** Summary of future research questions using the PICOS framework.

Domain	Research focus	P (Population)	I (Intervention)	C (Comparison)	O (Outcomes)	S (Study design)
Diagnostics	Use of advanced imaging (CBCT, MRI, intraoral scanning) in treatment planning	Patients with edentulous maxillae indicated for full‐arch implant rehabilitation	CBCT/MRI integrated with digital workflows	Conventional two‐dimensional radiographs or clinical exam only	Implant placement accuracy, detection of complications, radiation exposure, PROMs	RCTs, prospective cohort studies
Hard Tissue Augmentation	Effectiveness of different GBR materials and fixation methods	Patients requiring vertical/horizontal ridge augmentation before implant placement	GBR with biomaterials and fixation devices	GBR without fixation or using alternative biomaterials	Ridge gain, membrane exposure, complication rate, implant survival	RCTs, prospective case series, multicenter registries
Soft Tissue Augmentation	Need for keratinized mucosa around full‐arch implants	Patients rehabilitated with full‐arch prostheses and < 2 mm keratinized mucosa	Connective tissue graft or soft tissue substitutes	No augmentation	Keratinized mucosa width, peri‐implant health, mucositis incidence, PROMs (comfort, esthetics, ease of cleaning)	RCTs, network meta‐analyses, long‐term cohorts
Complication Management	Management of intraoperative complications during sinus or ridge augmentation	Patients undergoing sinus floor elevation or complex augmentation	Collagen membrane repair and continuation	Abandoning augmentation or staged approach	Complication resolution, implant survival, sinus pathology, patient morbidity	Prospective controlled studies, RCTs (where ethical)
Maintenance	Optimal recall and supportive peri‐implant therapy protocols	Patients with full‐arch implant prostheses	Supportive peri‐implant therapy with probing, radiographs, hygiene	Prosthetic maintenance only (no probing/radiographs)	Early detection of peri‐implantitis, bone stability, implant/prosthesis survival	Long‐term prospective studies, pragmatic RCTs
Maintenance	Impact of prosthesis retrieval and screw replacement during recall	Patients with screw‐retained full‐arch prostheses	Routine prosthesis removal and screw renewal	Prosthesis removal only in case of peri‐implantitis	Peri‐implant health, prosthesis longevity, complication rate	RCTs, multicenter prospective cohorts
Maintenance	Effectiveness of occlusal guards	Patients with implant‐supported full‐arch prostheses	Night guard use	No night guard	Implant/prosthesis survival, fracture/complication rates, PROMs	RCTs, prospective cohorts

Clinical decision‐making must be individualized and involve shared deliberation between clinician and patient (Lin, Brunello, et al. 2026), taking into account systemic health, local anatomical conditions, prosthetic requirements, esthetic demands, and patient expectations (e.g., fixed versus removable prostheses). Successful rehabilitation of the edentulous maxilla with implants requires clinicians to have the requisite expertise, training, and judgment. Treatment planning should integrate anatomical considerations (e.g., sinus pneumatization, ridge morphology), prosthetic needs (e.g., lip support, occlusion, esthetics), and the resources available, ensuring that outcomes align with both biological feasibility and patient‐centered goals (Brunello, Lin, et al. 2026; Brunello, Strauss, et al. 2026; Sabri et al. 2026; Saleh et al. 2026).

## 3 Methodological Framework and Consensus Procedures of the 1st GCCG

**FIGURE 1:**
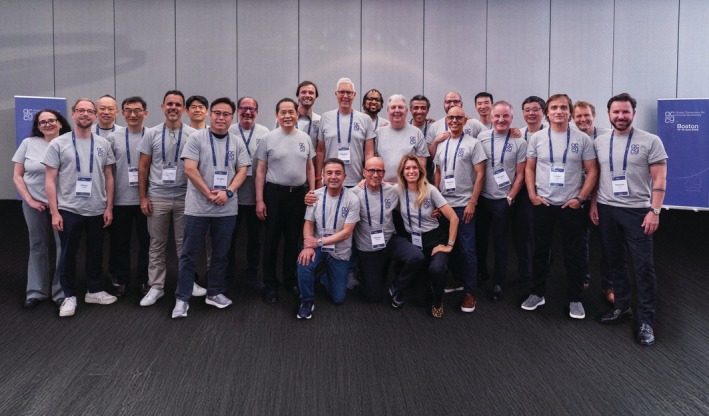
Participants of the Working Group 3 of the 1st Global Consensus for Clinical Guidelines (GCCG) for the rehabilitation of the edentulous maxilla (Boston, June 2025).

The Working Group 3 was one of four formed within the 1st Global Consensus Conference on Clinical Guidelines (GCCG) for the Rehabilitation of the Edentulous Maxilla, following an S2k‐level framework of the Association of the Scientific Medical Societies in Germany (AWMF). The Working Group 3 included 27 of the 105 participating experts (Figure 1). These 27 experts represented three specialties: prosthodontics (5), periodontics (13), and oral surgery (9).

Group recommendations were developed through a structured, multi‐phase process combining systematic evidence synthesis with input from expert clinicians as well as patient survey and cross‐disciplinary expert survey results. Preparatory work included systematic reviews on PROs and ClinROs, and their respective measures (Sabri et al. 2026; Saleh et al. 2026), as well as single‐round international surveys of clinicians, patients, and cross‐disciplinary experts (Brunello, Strauss, et al. 2026; Lin, Brunello, et al. 2026; Lin, Strauss, et al. 2026; Schoenbaum et al. 2026; Schwarz et al. 2026; Strauss et al. 2026). These data provided the foundation for Working Group discussions during the in‐person meeting in Boston (June 16–18, 2025).

Using the Nominal Group Technique, each recommendation was developed and refined through individual review, prioritization voting, and moderated discussion. Recommendations were primarily derived from the results of the structured surveys and reviewed in the context of the available evidence and categorized as

- Aligned with current evidence (survey results consistent with available data)
- Not aligned with current evidence (survey results diverged from available data) or
- Could not be evaluated due to a lack of existing evidence.

Final recommendations from the Working Group 3 were established through anonymous plenary voting, using predefined thresholds of agreement: strong consensus (> 95% agreement); consensus (≥ 75% but ≤ 95% agreement); no consensus (< 75% agreement). Conflicts of interest (CoI) were disclosed as per ICMJE standards and abstentions were documented. The full methodological framework is detailed in the umbrella paper (Schwarz et al. 2026).

## 4 Summary of the Reported Clinician‐ and Patient‐Reported Outcomes

In preparation for the 1st GCCG in Implant Dentistry, eight systematic reviews were conducted to identify and evaluate the available evidence on ClinROs and PROs, along with their respective outcome measures. This section summarizes the most relevant findings from the two systematic reviews (Sabri et al. 2026; Saleh et al. 2026) that informed the discussions of Working Group 3 with regard to ClinROs and PROs used in studies that evaluated alveolar ridge and sinus floor augmentation in the rehabilitation of the edentulous maxilla.

### 4.1 ClinROs/CROMs

Relevant ClinROs and CROMs were identified through structured data extraction from the primary studies included in two systematic reviews (Sabri et al. 2026; Saleh et al. 2026). These outcomes were grouped into the following categories:

**Clinical outcomes**

1. Implant survival: The most widely reported outcome (Boven et al. 2020; Carames et al. 2022; Kappel et al. 2021; Lie et al. 2021, 2024; Onclin et al. 2023; Putters et al. 2019; Rickert et al. 2014; Slot et al. 2016, 2023; Urban et al. 2017; Wortmann et al. 2021; Wu et al. 2015; Xavier et al. 2015), typically defined as the absence of mobility or loss. Some trials applied standardized criteria (e.g., Pisa consensus) (Albrektsson et al. 1986), while others defined survival pragmatically.
2. Implant success: Assessed in a subset of studies (Aly and Hammouda 2017; Castagna et al. 2013; Kappel et al. 2021; Lie et al. 2021; Mordenfeld et al. 2016; Wu et al. 2015), though definitions varied (clinical stability, absence of pain or radiolucency, limited bone loss, or consensus‐based criteria).
3. Prosthesis survival and success: Several studies reported overdenture survival rates (Boven et al. 2020; Grandi et al. 2019; Lie et al. 2021; Onclin et al. 2023; Slot et al. 2019, 2023), with failure defined as the need for replacement. Prosthetic success was variably defined (e.g., full functionality, absence of occlusal disturbances, or consensus criteria).
4. Plaque index: Plaque, calculus, and peri‐implant mucosal inflammation were reported (Boven et al. 2017, 2020; Kappel et al. 2021; Onclin et al. 2023; Rickert et al. 2014; Slot et al. 2016, 2019, 2023; Wu et al. 2015) using established indices (Loe 1967; Mombelli and Lang 1994).
5. Probing depth: Reported directly in a minority of studies (Boven et al. 2017, 2020; Onclin et al. 2023; Rickert et al. 2014; Rossi et al. 2021; Slot et al. 2016, 2019, 2023; Wu et al. 2015), usually from six sites per implant; in some trials, probing depth contributed indirectly to peri‐implant disease diagnosis (Berglundh et al. 2018).
6. Implant primary stability: Insertion torque and implant stability (via resonance frequency analysis) were reported in several studies (Aly and Hammouda 2017; Hernandez‐Alfaro et al. 2013; Markovic et al. 2022; Putters et al. 2019).

**Radiographic outcomes**

1. Radiographic marginal bone level: One of the most consistently reported imaging outcomes (Aludden et al. 2024; Aly and Hammouda 2017; Boven et al. 2017, 2020; Carames et al. 2022; Castagna et al. 2013; Grandi et al. 2019; Lie et al. 2021, 2024; Mordenfeld et al. 2016; Onclin et al. 2023; Putters et al. 2018, 2019; Rickert et al. 2014; Rossi et al. 2021; Slot et al. 2016, 2019, 2023; Urban et al. 2017; Wu et al. 2015), assessed with standardized periapical radiographs, panoramic images, or CBCT.
2. Sinus/bone augmentation outcomes: Multiple studies measured sinus floor elevation results or horizontal/vertical bone changes with CBCT or panoramic imaging (Castagna et al. 2013; Hernandez‐Alfaro et al. 2013; Lie et al. 2021, 2024; Mordenfeld et al. 2016; Urban et al. 2017; Wortmann et al. 2021).

**Complication outcomes**

1. Peri‐implant mucositis and peri‐implantitis: Incidence of peri‐implant mucositis and peri‐implantitis was reported in two studies (Putters et al. 2019; Slot et al. 2016).
2. Surgical/intraoperative and postoperative complications: Studies reported intra‐ and post‐operative events (Carames et al. 2022; Castagna et al. 2013; Grandi et al. 2019; Markovic et al. 2022; Putters et al. 2018, 2019; Rossi et al. 2021; Slot et al. 2023; Urban et al. 2017), such as sinus membrane perforation, flap dehiscence, infection, or donor‐site morbidity.
3. Prosthodontic maintenance events/complications: Included technical issues such as wear of retention components, replacement of attachments, or denture adjustments for pressure spots (Kappel et al. 2021; Slot et al. 2016; Wu et al. 2015).

### 4.2 PROs/PROMs

Across the included studies, approximately half reported PROs and PROMs, which capture patients' perspectives on chewing function, satisfaction, and quality of life. Several validated instruments and custom scales were employed:

1. Quality of life (assessed for example with Oral Health Impact Profile [OHIP]): Variants of OHIP (OHIP‐49, OHIP‐49NL, OHIP‐EDENT‐19, and OHIP‐20E) were the most frequently used tools (Boven et al. 2020; Lie et al. 2024; Markovic et al. 2022; Onclin et al. 2023; Rossi et al. 2021). These questionnaires assess oral health‐related quality of life across multiple domains, with shorter versions tailored for edentulous patients.
2. Denture Satisfaction Questionnaire (DSQ): Introduced by Vervoorn et al. (1988), this validated instrument measures patient satisfaction across domains such as function, esthetics, and comfort (Boven et al. 2017, 2020; Onclin et al. 2023; Slot et al. 2016, 2019).
3. Chewing function: Used in multiple trials (Boven et al. 2017; Lie et al. 2024; Onclin et al. 2023; Slot et al. 2016, 2019, 2023) to assess patients' perceived ability to chew different types of foods (Stellingsma et al. 2005).
4. Pain: Visual Analogue Scale (VAS) widely applied to measure pain (Aly and Hammouda 2017; Markovic et al. 2022; Putters et al. 2019; Wortmann et al. 2021), typically on a 0–10 scale (Miller and Ferris 1993).
5. Patient overall satisfaction with treatment: Some studies used single‐item 10‐point scales to assess global denture or treatment satisfaction (Boven et al. 2020; Rickert et al. 2014; Slot et al. 2023).

Overall, PROMs consistently highlighted treatment‐related improvements in quality of life, satisfaction, and functional ability, though heterogeneity in measurement tools limited direct comparability across studies. The systematic reviews available to the Working Group 3 (Sabri et al. 2026; Saleh et al. 2026) confirmed that overall oral health–related quality of life (OHRQoL) is one of the most frequently used measures for evaluating outcomes in the rehabilitation of the edentulous maxilla. OHRQoL is associated with high patient satisfaction, regardless of specific techniques employed, as long as treatment is successful. However, there was considerable heterogeneity in outcome reporting among studies. Many studies employed non‐standardized or non‐validated questionnaires for PROs, and even when validated tools were used, they were not always specifically designed for edentulous maxilla rehabilitation, complicating comparisons across studies. There was also minimal reporting on whether outcome assessors were calibrated or whether PROMs were administered consistently, indicating room for improvement in future research methodology.

During the in‐person discussion of Working Group 3, although many studies reported similar objective ClinROs and PROs, their definitions varied substantially, particularly for complex parameters such as treatment success. Following the three rounds of the onsite Delphi process to define a Core Outcome Set (COS) for edentulous maxilla rehabilitation (Brunello, Lin, et al. 2026), one PRO (pain) and one ClinRO (implant primary stability) reached consensus relevance (≥ 75% agreement) among the participants (Brunello, Lin, et al. 2026). For complications, one PRO (complications during treatment/maintenance) and eight ClinROs (prosthodontic maintenance events/complications, implant failure, prosthesis failure, mechanical/technical complications, prosthetic complications, postoperative complications, peri‐implant suppuration, and peri‐implantitis) were considered relevant (≥ 75% agreement) (Brunello, Lin, et al. 2026). A universal outcome set is still lacking, emphasizing the need for harmonization. The Working Group 3 recommends validated tools such as OHIP‐14 for quality of life and standardized peri‐implant indices for clinical evaluation, along with surgical parameters (e.g., bone gain, sinus membrane perforation) and long‐term metrics (e.g., peri‐implant soft tissue stability, peri‐implantitis incidence, prosthesis maintenance).

## 5 Summary of the Key Findings from the Surveys

To complement the evidence synthesized from systematic reviews, three single‐round surveys were conducted before the in‐person GCCG meeting. The first involved 217 invited experts from 43 countries, achieving a 53.5% response rate (Brunello, Strauss, et al. 2026). The second surveyed 68 invited patients, and the third included 68 cross‐disciplinary experts, with response rates of 60% and 31%, respectively (Lin, Brunello, et al. 2026). The objective was to identify prevailing practices, preferences, and areas of consensus regarding the patient selection, diagnostics, treatment planning, treatment procedures, and maintenance of implant‐supported rehabilitations in the edentulous maxilla. The key findings from the surveys are summarized below.

### 5.1 Patient Selection

In cases of severe maxillary atrophy, 55.1% of responded experts favored the use of standard‐length implants with sinus augmentation or bone grafting, rather than short implants or zygomatic implants. When adequate anterior maxillary bone was present, 50.0% preferred implant placement confined to this region without grafting.

Patients reported that they felt well‐informed by their dentists regarding treatment expectations and the anticipated longevity of implants. Similarly, cross‐disciplinary experts also agreed that these aspects—treatment expectations and expected implant longevity—should be clearly communicated in clinical practice.

### 5.2 Diagnostics

Strong consensus (> 95%) was reached on the need for preoperative sinus assessment in cases involving sinus lifts (lateral: 96.6%; crestal: 90.6%) or suspected pathology (95.8%). Consensus was also achieved for zygomatic implants (91.4%), but not for short implants (66.0%) or standard‐length implants in native bone (61.8%).

CT/CBCT scans were strongly recommended (96.5%). Consensus was reached for intraoral scans, photographs, wax‐ups, and mock‐ups, while panoramic x‐rays (67.8%) and facial scans (56.8%) did not achieve consensus. No consensus emerged on impression methods, though 67.8% supported conventional impressions, reflecting a shift toward digital approaches (Brunello, Strauss, et al. 2026; Lin, Brunello, et al. 2026).

Most patients reported undergoing CBCT scans before implant placement, and cross‐disciplinary experts concurred that CBCT imaging is advisable prior to rehabilitating the edentulous maxilla. While patients generally expressed little concern about radiation, some cross‐disciplinary experts raised issues, especially with multiple scans. Both groups showed no strong preference for impression techniques, though digital methods were slightly favored.

### 5.3 Treatment Planning

Experts showed considerable variability when specifying the minimum subantral bone height required for simultaneous implant placement with sinus lift procedures. For the lateral approach, most recommended 3–4 mm, although a small proportion considered 1 mm (5.2%) or 2 mm (8.6%) acceptable. For the crestal approach, the most frequent threshold was 5 mm (44.8%), followed by 4 mm (20.7%) and 6 mm (19.8%).

In cases of multiple implant placement in the fully edentulous maxilla, no consensus was reached on whether freehand, static, or dynamic guided surgery should be preferred.

### 5.4 Treatment Procedures

In posterior maxilla cases requiring sinus lift or bone grafting, 76.7% of experts favored delayed implant placement over immediate or early placement. Reported healing intervals before second‐stage surgery ranged from 2 to 10 months, most commonly 6 months (43.1%) or 4 months (19%), but no consensus emerged on the optimal timing. Immediate loading after sinus lift or grafting with good primary stability was generally discouraged. Additionally, 85.4% of survey respondents disagreed with non‐splinted provisional restorations, and no consensus was reached on splinted provisionals, though most experts remained cautious.

No overall consensus was reached on preferred grafting materials. For lateral and crestal sinus lifts with simultaneous implant placement, xenogeneic substitutes were most frequently used, while combinations of biomaterials were preferred for sinus lift and other augmentation procedures with delayed implant placement. Consensus was reached on the need of fixing barrier membranes (pins, sutures, or other methods) to achieve favorable clinical outcomes in vertical bone augmentation. Additionally, there was agreement on the negative impact of titanium mesh and non‐resorbable membrane exposure on bone regeneration. The effect of resorbable membrane exposure remained inconclusive. No consensus was reached on the use of biologics, including blood concentrates, either alone or as barrier membranes; however, some agreement (60.3%) supported combining biologics with bone substitutes.

For cases with insufficient keratinized mucosa, 83.6% of experts agreed that soft tissue augmentation is typically performed either at implant placement or during second‐stage surgery to establish keratinized mucosa.

In cases of sinus membrane perforation during lateral sinus lift, expert recommendations varied by perforation size. For perforations ≤ 5 mm, experts recommended continuing the surgery without interruption (76.7%) while strongly supporting use of resorbable membranes for repairing the perforation (94.8%). For perforations between 5 and 10 mm, collagen membranes were recommended by 84.4% of patients. For perforations > 10 mm, bone blocks were generally considered inappropriate (76.7%).

Preferences for interim prostheses in fully edentulous ridges varied widely. About 20% advised avoiding prostheses during early healing, > 25% favored temporary implant‐supported restorations, while others recommended soft relined dentures or removable flangeless prostheses.

Routine antibiotic use during full‐arch implant placement did not reach consensus. Still, 68.1% of experts reported always prescribing prophylactic antibiotics, and 26.7% prescribed selectively (e.g., for medically compromised patients, sinus lift, GBR, or bone block procedures). After implant insertion, 68.9% routinely prescribed antibiotics and 27.6% did so only in specific cases.

### 5.5 Maintenance

Consensus was reached on the value of performing full‐mouth pocket charting (85.4%) and intraoral radiographs (78.5%) at least once per year during long‐term follow‐up in the absence of complications. By contrast, most experts disagreed with the routine use of CBCT (77.7%), and no consensus was achieved on the regular use of panoramic radiographs during the maintenance phase.

Experts generally expect implants placed in native bone to last over 10 years without complications (87.1%), with similar expectations for implants placed with lateral or crestal sinus lifts (76.7% and 78.4%, respectively). Although a majority also anticipated a lifespan exceeding 10 years for implants with GBR (65.5%) or bone blocks (59.5%), no consensus was reached for these techniques.

Experts did not reach a consensus on the routine use of the listed emerging materials and techniques for rehabilitating the fully edentulous maxilla over the next 5 years. These include dynamic and robotic‐guided surgery, three‐dimensional (3D) printed scaffolds and meshes, and stem cells. Notably, more than 40% of the experts disagreed with the idea of widespread adoption of robotic surgery and stem cells for this indication within this timeframe.

For maxillary full‐arch rehabilitation with implants requiring sinus or ridge augmentation, experts agreed that chewing function and phonetics are key short‐term determinants of patient satisfaction (> 90% agreement). No consensus was reached regarding treatment selection based on available evidence (27.6%), technical difficulty (69.8%), or perceived procedural ease (71.6%).

## 6 Working Group 3 Consensus Recommendations

**Recommendation No. 1—Diagnostic Tools and Treatment Planning (Aligned with current evidence)**.

**Table:** 

Category	Details
Domain	Diagnostic Tools & Treatment Planning
Recommendation	For patients with a fully edentulous maxilla undergoing implant rehabilitation, three‐dimensional imaging (CBCT or medical CT) should be used routinely for preoperative evaluation and digital planning. This enables accurate assessment of bone volume, recognition of anatomical constraints, and prosthetically driven implant positioning (Harris et al. 2012; Katsoulis et al. 2009; Valentini and Artzi 2023)
Expert Survey Results	Experts overwhelmingly reported routine use of CBCT. Patients and cross‐disciplinary experts generally expected CBCT when the risks and benefits were clearly explained
Cross‐disciplinary Experts and Patient Survey Results	Patients expressed some concern about radiation exposure but reported high overall acceptance when CBCT was explained as clinically necessary. Cross‐disciplinary experts emphasized the importance of justification for radiation exposure
Supporting/Contradicting Literature	Guidelines recommend cross‐sectional imaging for guided surgery, augmentation planning, and management of complex anatomy (Harris et al. 2012). Digital planning has been shown to improve prosthetically driven implant placement (Katsoulis et al. 2009), and narrative reviews support CBCT as essential for edentulous maxilla rehabilitation (Valentini and Artzi 2023)
Recommended ClinROs/CROMs	Implant survival Implant success Prosthesis success Clinician's treatment success Surgical/intraoperative complications Implant primary stability
Recommended PROs/PROMs	Patient overall satisfaction with treatment Chewing function/comfort/discomfort Complications during treatment/maintenance Speech/phonetics/pronunciation function Ease of cleaning/oral hygiene efficacy Quality of life (Oral Health‐Related Quality of Life, OHRQoL)
Strength of Consensus	Agree: 81% (Consensus) Agree: 71/Disagree: 14/Abstain: 1/Abstain (CoI): 2

**Recommendation No. 2—Hard Tissue Augmentation (Aligned with current evidence)**.

**Table:** 

Category	Details
Domain	Hard Tissue Augmentation
Recommendation	For vertical ridge augmentation in the edentulous maxilla, barrier membranes should be securely fixed (tacks, pins, sutures, or screws) to maintain space and limit micromotion, thereby improving predictability and reducing exposure risk (Wang and Boyapati 2006; Wessing et al. 2018)
Expert Survey Results	The large majority of experts supported the use of membrane fixation, citing improved stability and a reduction in complications
Cross‐disciplinary Experts and Patient Survey Results	Not directly applicable, as patients are typically unaware of fixation methods. Cross‐disciplinary experts, however, emphasized the value of predictable outcomes and reduced complication rates
Supporting/Contradicting Literature	The PASS principles highlight the importance of stability and space maintenance (Wang and Boyapati 2006). Meta‐analyses further suggest that fixation enhances vertical bone gain (Wessing et al. 2018)
Recommended ClinROs/CROMs	Implant survival Implant success Implant primary stability Radiographic marginal bone loss Biological complications Surgical/intraoperative complications Postoperative complications Peri‐implant health Peri‐implant mucositis Peri‐implantitis
Recommended PROs/PROMs	Complications during treatment/maintenance Pain Patient overall satisfaction with treatment Patient‐reported complaints Aesthetic satisfaction Quality of life (Oral Health‐Related Quality of Life, OHRQoL)
Strength of Consensus	Agree: 80% (Consensus) Agree: 66/Disagree: 9/Abstain: 4/Abstain (CoI): 3

**Recommendation No. 3—Soft Tissue Augmentation (Aligned with current evidence)**.

**Table:** 

Category	Details
Domain	Soft Tissue Augmentation
Recommendation	In patients with insufficient keratinized mucosa, soft tissue augmentation should be performed at implant placement or at second stage to improve peri‐implant health and facilitate oral hygiene (Covani et al. 2013; Lin et al. 2013; Stefanini et al. 2023; Tavelli et al. 2021; Thoma et al. 2018)
Expert Survey Results	There is consensus that maintaining a minimal band of keratinized mucosa improves peri‐implant health and patient comfort
Cross‐disciplinary Experts and Patient Survey Results	Patients report improved hygiene and comfort following keratinized mucosa augmentation, and cross‐disciplinary experts associate adequate keratinized mucosa with a reduced incidence of mucositis
Supporting/Contradicting Literature	Systematic reviews and meta‐analyses confirm that soft tissue augmentation increases keratinized mucosa width and enhances peri‐implant health indices (Covani et al. 2013; Lin et al. 2013; Stefanini et al. 2023; Tavelli et al. 2021; Thoma et al. 2018)
Recommended ClinROs/CROMs	Implant survival Implant success Radiographic marginal bone loss Biological complications Surgical/intraoperative complications Postoperative complications Width of keratinized mucosa Plaque index/oral hygiene Peri‐implant health Peri‐implant mucositis Peri‐implantitis
Recommended PROs/PROMs	Complications during treatment/maintenance Ease of cleaning/oral hygiene efficacy Pain Patient overall satisfaction with treatment Aesthetic satisfaction Quality of life (Oral Health‐Related Quality of Life, OHRQoL)
Strength of Consensus	Agree: 91% (Consensus) Agree: 62/Disagree: 4/Abstain: 1/Abstain (CoI): 1

**Recommendation No. 4—Management of Intraoperative Complications (Sinus Membrane Perforation) (Aligned with current evidence)**.

**Table:** 

Category	Details
Domain	Intraoperative Complication Management (Sinus Membrane Perforation)
Recommendation	If a Schneiderian membrane perforation ≤ 10 mm occurs during sinus augmentation, the perforation can be covered with a resorbable collagen membrane and augmentation can proceed. Tears < 5 mm: strong recommendation to continue. Tears 5–10 mm: suggest continuation with careful technique. Larger tears may require staging or aborting the procedure (Diaz‐Olivares et al. 2021; Hernandez‐Alfaro et al. 2008; Khoury et al. 2024; Molina et al. 2022; Pjetursson and Lang 2014; Raghoebar et al. 2019; Testori et al. 2023; Wallace and Froum 2003)
Expert Survey Results	94.8% supported repair and continuation for ≤ 5 mm tears; 84.4% supported continuation for 5–10 mm tears, with some exercising more caution
Cross‐disciplinary Experts and Patient Survey Results	Not directly applicable, as patients are generally unaware of intraoperative events. Indirect relevance lies in preventing delays and complications
Supporting/Contradicting Literature	Collagen‐based repair of perforations has been shown to yield bone regeneration and implant survival rates comparable to non‐perforated cases (Diaz‐Olivares et al. 2021; Hernandez‐Alfaro et al. 2008; Pjetursson and Lang 2014; Wallace and Froum 2003). However, data remain limited for very large tears
Recommended ClinROs/CROMs	Implant survival Implant success Radiographic marginal bone loss Surgical/intraoperative complications Biological complications Postoperative complications Peri‐implant health
Recommended PROs/PROMs	Complications during treatment/maintenance Pain Patient overall satisfaction with treatment Patient‐reported complaints
Strength of Consensus	Agree: 82% (Consensus) Agree: 64/Disagree: 8/Abstain: 4/Abstain (CoI): 2

## 7 Summary of Areas for Future Research

Despite advances in implant therapy for the edentulous maxilla, several clinical questions remain regarding the optimal indications and approaches for bone augmentation. One key area for future investigation is the timing and criteria for sinus augmentation in the treatment of the severely atrophic maxilla. While sinus augmentation is generally indicated when residual vertical bone height is insufficient and ridge width is adequate, the thresholds for when short implants may suffice versus when sinus augmentation becomes necessary remain unclear. Additionally, the influence of the anterior–posterior dimension of the maxillary sinus on implant positioning and prosthetic support warrants further study, particularly in relation to PROs such as prosthesis function and morbidity.

Similarly, the indications for ridge augmentation require additional evidence. Horizontal ridge augmentation is typically recommended when ridge width is insufficient for implant placement; however, the interplay between ridge augmentation and sinus anatomy in optimizing implant site development has not been fully established. Specifically, research is needed to determine whether limited anterior ridge augmentation between the right and left sinuses can reliably eliminate the need for lateral sinus augmentation, thereby reducing patient morbidity and complication risk while still allowing adequate prosthetic rehabilitation.

Although less commonly performed, vertical ridge augmentation remains an important area for future research. Its indications are generally limited to severe localized vertical deficiencies, often combined with horizontal augmentation. Further evidence is needed to determine when vertical augmentation provides meaningful improvements in implant outcomes relative to patient morbidity, surgical complexity, and alternative treatment options. Additionally, some techniques, such as the split bone block approach (Khoury and Hanser 2015, 2019), were not fully explored in the surveys. This technique involves harvesting autologous bone blocks from the mandibular symphysis or ramus using piezoelectric surgery or microsaws, splitting each block into two thin laminae with microscrews, and filling the intervening gap with autogenous bone chips. Future studies should evaluate both ClinROs and PROs associated with these approaches.

Finally, the decision‐making process between lateral window sinus augmentation and transcrestal sinus elevation requires further study. While transcrestal approaches may be appropriate when residual bone height is approximately 4–5 mm, lateral window techniques are often preferred when residual bone is < 4 mm or when multiple implants are planned in the posterior maxilla. Comparative research evaluating clinical outcomes, complication rates, and PROMs for these two approaches will help refine treatment algorithms and enhance patient‐centered care. Future research should also evaluate alternative methods for repairing sinus membrane perforations, including the use of tissue glue or suturing techniques.

## 8 Conclusions

The consensus guidelines developed by the Working Group 3 provide a structured framework for advanced diagnostics and therapeutic interventions in the rehabilitation of the edentulous maxilla. The recommendations integrate current evidence and international consensus on 3D imaging, hard and soft tissue augmentation, and complication management, with the aim of enhancing predictability and long‐term clinical outcomes in full‐arch maxillary implant therapy. While these guidelines provide a valuable reference for clinical decision‐making, several areas remain underpinned by limited or heterogeneous evidence. Ongoing prospective studies and high‐quality clinical trials are necessary to validate these approaches, address existing gaps, and ensure that future iterations of the guidelines remain aligned with emerging technologies and patient‐centered care.

## Author Contributions

**Joseph Fiorellini, Guo‐Hao Lin, Isabella Rocchietta, Sean Mojaver, Christer Dahlin:** writing – original draft. **Joseph Fiorellini, Guo‐Hao Lin, Hom‐Lay Wang** and participants of Working Group 3: writing – review and editing. **Hom‐Lay Wang:** conceptualization. **Guo‐Hao Lin, Hom‐Lay Wang:** methodology. **Hom‐Lay Wang:** supervision. All contributors and Working Group 3 participants: Approval of manuscript.

## Funding

This work was supported by the European Association for Osseointegration, International Team for Implantology, and Osteology Foundation.

## Conflicts of Interest

All delegates disclosed secondary interests using the standardized ICMJE disclosure form. Potential conflicts of interest (CoI) were actively managed in accordance with Guidelines International Network (GIN) principles.

## Data Availability

The data that support the findings of this study are available from the corresponding author upon reasonable request.
